# Distinct patterns of white matter hyperintensity and cortical thickness of *CSF1R*-related leukoencephalopathy compared with subcortical ischemic vascular dementia

**DOI:** 10.1371/journal.pone.0308989

**Published:** 2024-10-07

**Authors:** Seung Joo Kim, Wanzee Cho, Hee Jin Kim, Duk L. Na, Sang Won Seo, Na-Yeon Jung, Jae-Hyeok Lee, Myung Jun Lee, Heeyoung Kang, Joon-Kyung Seong, Eun-Joo Kim

**Affiliations:** 1 Department of Neurology, Gyeongsang National University School of Medicine and Gyeongsang National University Changwon Hospital, Changwon, South Korea; 2 Department of Neurology, Samsung Medical Centre, Sungkyunkwan University School of Medicine, Seoul, South Korea; 3 Department of Artificial Intelligence, Korea University, Seoul, South Korea; 4 Alzheimer Disease Convergence Research Centre, Samsung Medical Centre, Seoul, South Korea; 5 Departments of Health Sciences and Technology and, Digital Health, SAIHST, Sungkyunkwan University, Seoul, South Korea; 6 Department of Neurology Pusan National University Yangsan Hospital, Pusan national University School of Medicine, Yangsan, South Korea; 7 Research Institute for Convergence of Biomedical Science and Technology, Pusan National University Yangsan Hospital, Pusan national University School of Medicine, Yangsan, South Korea; 8 Department of Neurology, Pusan National University Hospital, Pusan National University School of Medicine and Medical Research Institute, Busan, South Korea; 9 Department of Neurology, Gyeongsang National University Hospital, Jinju, South Korea; 10 School of Biomedical Engineering, Korea University, Seoul, South Korea; University of Rochester, UNITED STATES OF AMERICA

## Abstract

**Background:**

*CSF1R*-related leukoencephalopathy is a type of autosomal dominant leukodystrophy caused by mutations in the colony stimulating factor 1 receptor (*CSF1R*) gene. Subcortical ischemic vascular dementia (SIVaD), which is caused by cerebral small vessel disease, is similar to *CSF1R*-related leukoencephalopathy in that it mainly affects subcortical white matter. In this study, we compared the patterns of white matter hyperintensity (WMH) and cortical thickness in *CSF1R*-related leukoencephalopathy with those in SIVaD.

**Methods:**

Fourteen patients with *CSF1R*-related leukoencephalopathy and 129 with SIVaD were retrospectively recruited from three tertiary medical centers. We extracted and visualized WMH data using voxel-based morphometry to compare the WMH distributions between the two groups. Cortical thickness was measured using a surface-based method. Statistical maps of differences in cortical thickness between the two groups were generated using a surface model, with age, sex, education, and intracranial volume as covariates.

**Results:**

Predominant distribution of WMH in the *CSF1R*-related leukoencephalopathy group was in the bilateral frontal and parietal areas, whereas the SIVaD group showed diffuse WMH involvement in the bilateral frontal, parietal, and temporal areas. Compared with the SIVaD group, the *CSF1R*-related leukoencephalopathy group showed more severe corpus callosum atrophy (CCA) and widespread cortical thinning.

**Conclusions:**

To our knowledge, this is the first study using the automated MR measurement to capture WMH, cortical thinning, and CCA with signal changes in *CSF1R*-related leukoencephalopathy. It provides new evidence regarding differences in the patterns of WMH distribution and cortical thinning between *CSF1R*-related leukoencephalopathy and SIVaD.

## Introduction

*CSF1R*-related leukoencephalopathy, previously known as adult-onset leukoencephalopathy with axonal spheroids and pigmented glia, is a rare autosomal dominant white matter disease caused by mutations in the colony stimulating factor 1 receptor (*CSF1R*) gene [1–4]. It has variable manifestations including cognitive decline, behavioral changes, motor dysfunction, and seizures [4–6]. As the disease progresses, patients eventually become bedridden with spasticity and rigidity [7]. Pathologically, *CSF1R*-related leukoencephalopathy is characterized by diffuse white matter degeneration and widespread axonal spheroids [2] observable as bilateral white matter lesions, cortical atrophy, a thinner corpus callosum (CC), and dilation of the lateral ventricles on magnetic resonance imaging (MRI) [1].

Given the heterogeneous clinical symptoms and characteristic but non-specific radiological findings of leukoencephalopathy, *CSF1R*-related leukoencephalopathy is often misdiagnosed as various other white matter diseases, including vascular dementia (VaD), primary progressive multiple sclerosis, other adult-onset leukodystrophies, toxic or metabolic disease, and inflammatory white matter diseases [8, 9]. Of these, VaD, especially subcortical ischemic vascular dementia (SIVaD), which is caused by cerebral small vessel disease, is the most common disease involving subcortical white matter and is also characterized by cognitive decline with motor deficits [10–12]. It is associated with vascular risk factors such as hypertension, diabetes mellitus or dyslipidemia [9]. Early detection of SIVaD is clinically important because managing these vascular risk factors can prevent its progression [13]. Alternatively, although microglia replacement by hematopoietic stem cell transplantation has been attempted [14], or a phase 2 study of VGL101 as a high-affinity human TREM2 agonistic monoclonal antibody (ClinicalTrials.gov ID NCT05677659) is ongoing, currently there are no approved disease-modifying therapy for *CSF1R*-related leukoencephalopathy. So an accurate and timely diagnosis is crucial for patients to save the extra money for extensive work-up and plan for long-term supportive therapy, and for physicians to optimize genetic counselling for family members.

A white matter imaging approach might be helpful in differentiating between *CSF1R*-related leukoencephalopathy and SIVaD, because both diseases are representative brain white matter diseases. Therefore, we compared the regional distributions of white matter hyperintensity (WMH) and changes in cortical thickness in *CSF1R*-related leukoencephalopathy with those in SIVaD to identify the patterns specific to the former using automated approaches. Although numerous neuroimaging studies on *CSF1R*-related leukoencephalopathy have been reported, this is the first study to automatically quantify WMH and measure cortical thinning. Even if this automated imaging approach may not be routinely applied in a clinical setting, we believe that the inferences drawn from the results of the present study will be helpful for differential diagnosis.

## Methods

### Participants

This study was approved by the institutional review boards of Samsung Medical Center (IRB approval no. 2019-12-034), Pusan National University Hospital (IRB approval no. H-1911-023-085), and Pusan National University Yangsan Hospital (IRB approval no. 05-2016-081). We obtained written consent from each patient. This study was conducted from December 27, 2019 and all data with fully anonymized were drawn from retrospective cohorts. Fourteen patients with *CSF1R*-related leukoencephalopathy and 129 with SIVaD were retrospectively recruited from three tertiary medical centers (Samsung Medical Center, Pusan National University Hospital, and Pusan National University Yangsan Hospital) between February 2009 and October 2022. All patients with *CSF1R*-related leukoencephalopathy were genetically confirmed. 3 out of 14 patients with *CSF1R*-related leukoencephalopathy were also pathologically confirmed. SIVaD was clinically diagnosed using Diagnostic and Statistical Manual of Mental Disorders-Fourth Edition (DSM-IV) criteria for VaD and the following modified imaging requirements of the Fazekas ischemia and Erkinjuntti et al. criteria [14, 15]: (1) severe WMH on T2-weighted or fluid-attenuated inversion recovery (FLAIR) images (defined as periventricular WMH ≥ 10 mm and deep WMH ≥ 25 mm), and (2) deep grey matter lacunes. Patients with structural lesions including territorial cerebral infarction, brain tumors, WMH due to radiation injury, or multiple sclerosis, were excluded. They did not perform any genetic tests. For the cortical thickness and the mean volume of the CC analyses, 121 cognitively normal (CN) controls (mean age: 71.8 ± 7.0 years, male:female ratio, 47:74) with no history of neurological or psychiatric illness were selected from a cohort of volunteers at the Samsung Medical Center. All patients and CN controls underwent clinical evaluation, formal neuropsychological testing, and high-resolution 3.0-Tesla brain MRI (1 mm thickness T1-weighted and 2 or 5 mm thickness FLAIR imaging). Mean time intervals between disease onset and MRI study were 1.4 ± 1.7 years in *CSF1R*-related leukoencephalopathy and 3.6 ± 3.0 years in SIVaD groups, respectively. Mean durations between the neuropsychological tests and MRI of the patients with *CSF1R*-related leukoencephalopathy and SIVaD were 1.4 ± 2.7 months and 3.9 ± 10.5 months, respectively.

### Image acquisition and processing

We acquired standardized 3D T1-weighted and FLAIR images from all patients using MRI scanners (Philips Achieva, the Netherlands; Siemens Skyra, Germany; Siemens Verio and Magnetom Avanto, Germany) at the three participating centers. The detailed MRI parameters for each center are described in S1 Table.

### Assessment of WMH pattern

WMH data were extracted from FLAIR and T1-weighted images using the two Lesion Segmentation Tool (LST) algorithms. First, we used the Lesion Prediction Algorithm (LPA) [16] of the LST, which only requires FLAIR images and no additional parameters. The LPA results were compared with the actual FLAIR images, and if the extracted WMH values was different from the actual values, the other LST algorithm, the Lesion Growth Algorithm (LGA) [17], was used, which requires both FLAIR and T1-weighted images of a patient. We also had to input parameters and visually inspect the divisions produced by the different parameters and then use the optimal values determined in this manner. As the output of the LGA is in the T1 space, we linearly co-registered it to the FLAIR space using a Functional Magnetic Resonance Imaging of the Brain (FMRIB) software library (FMRIB’s Linear Image Registration Tool [18]) and nearest-neighbor interpolation. Additionally, to compare the WMH patterns between the *CSF1R*-related leukoencephalopathy and SIVaD groups, all WMH images were registered into Montreal Neurological Institute space, and significant WMHs were marked by color grade.

### Measurement of WMH volume

Among the results obtained through the FreeSurfer process, automated segmentation and classification of WMH was used to create a mask for each patient. Then, the overlapping regions of each patient’s mask and WMH were identified and the corresponding areas were calculated. WMH volumes of 68 cortical regions of interests (ROIs) and five ROIs corresponding to the CC were then acquired.

### Cortical thickness analyses

The publicly accessible, surface-based FreeSurfer package [19] was used for cortical thickness analysis. The analysis process included intensity normalization, registration to the Talairach space, exclusion of non-brain tissue, white matter segmentation, white matter boundary tessellation, smoothing of the tessellated surface, and automated topology correction. The deformable surface algorithm uses the tessellated surface as its starting point to delineate the white matter and then the pial boundary. The average distance from the white matter surface to the closest point on the pial surface and from that point back to the closest point on the white matter surface is used to determine the cortical thickness for each location on the tessellated white matter surface [20]. For all subjects, volumes were visually examined for reconstruction-related misclassifications after automated reconstruction. The recon-all script in FreeSurfer version 6.0 was used to obtain cortical thickness values for each T1-weighted scan. FreeSurfer output files were used to determine the thicknesses for all 68 ROIs of the Desikan–Killiany cortical parcellation. Additionally, the cortical thickness of 5 ROIs in the CC was also obtained resulting from FreeSurfer recon-all. Finally, the average CC volume was calculated using volume of genu, body and splenium from the result obtained through segmentation.

### Neuropsychological tests

All patients underwent standardized neuropsychological testing using the Seoul Neuropsychological Screening Battery (SNSB) [21]. This battery consists of several tests that assess performance across five domains: attention, language and related functions, visuospatial functions, memory, and executive functions. Scores were considered abnormal when the result was lower than the 16th percentile for age- and education-matched norms. As the age of the norms ranged from 45 to 90 years, two patients with *CSF1R*-related leukoencephalopathy who were below 45 years of age were matched with 45-year-old norms.

Based on the neuropsychological test results, we used the following quantitatively scorable tests for each domain: the digit span backward task for attention, the Korean version of the Boston Naming Test (K-BNT) [22] for language and related functions, the Rey–Osterrieth Complex Figure Test (RCFT; copy) for visuospatial functions, the Seoul Verbal Learning Test (SVLT; a 20 min delayed recall trial of 12 items) for memory, and the Stroop Test (color reading of 112 items during a 2 min period) for executive functions. Z-scores for the subtest in each domain were used for comparing cognitive performance between the *CSF1R*-related leukoencephalopathy and SIVaD groups and for correlation analysis between WMH volume and cognitive impairment.

### Statistical analyses

To compare the demographic and clinical variables of the *CSF1R*-related leukoencephalopathy and SIVaD groups, the Mann–Whitney U test (for continuous variables) and Fisher’s exact test (for categorical variables) were used. To evaluate the effects of regional WMH volume on cognitive outcomes in the *CSF1R*-related leukoencephalopathy group, multiple linear regression analysis was performed after controlling for age, sex, and education. SPSS version 24.0 software was used for all statistical analyses (SPSS Inc., Chicago, IL, USA).

WMH volume between the *CSF1R*-related leukoencephalopathy and SIVaD groups was compared using the Mann–Whitney U test. Frequency maps of WMH represented a considerably larger portion from the comparison between the two groups. Brain regions with a *p*-value of 0.05 or less were marked on the atlas in a color (ranging from yellow to red) corresponding to the *p*-value.

Localized differences in cortical thicknesses of the 68 cortical ROIs between the *CSF1R*-related leukoencephalopathy and SIVaD groups were examined using the Kruskal-Wallis test. Statistical maps of the differences in cortical thickness between the two groups were generated using a surface model, with age, sex, education, and intracranial volume as covariates. Only ROIs with *p*-values less than 0.05, as obtained using the ANCOVA, were considered as regions with significantly different cortical thickness.

## Results

### Demographic and clinical characteristics

The baseline demographic and clinical characteristics of the patients are presented in Tables 1 and 2. The mean age was significantly lower (*p* < 0.001) and educational level was significantly higher (*p* = 0.004) in the *CSF1R*-related leukoencephalopathy group compared with those in the SIVaD group. General cognitive indices, that is the Mini-Mental State Examination (MMSE) and Clinical Dementia Rating (CDR) scores, did not differ significantly between the two groups. With regard to the neuropsychological tests, only the visuospatial functions domain score was significantly lower in the *CSF1R*-related leukoencephalopathy group than in the SIVaD group (*p* = 0.013).

**Table 1:** Demographic and clinical characteristics of the included patients.

	*CSF1R*-related leukoencephalopathy (*N* = 14)	SIVaD (*N* = 129)	*p*-value
Age (mean ± SD), years	49.79 ± 6.13	76.05 ± 7.25	<0.001
Female, *N* (%)	7 (50%)	96 (74.4%)	0.065*
Education (mean ± SD), years	12.43 ± 5.16	7.97 ± 5.39	0.004
Interval between onset and MRI, years	1.38 ± 1.66	3.59 ± 2.96	0.004
Interval between MRI and neuropsychological tests, months	1.38 ± 2.67	3.93 ± 10.53	0.453
Neuropsychological test results (z-score)			
Attention	-1.56 ± 0.96	-1.19 ± 1.28	0.461
Language and related functions	-2.41 ± 1.77	-1.99 ± 1.87	0.568
Visuospatial functions	-15.32 ± 9.25	-3.25 ± 3.13	0.013
Memory	-2.43 ± 1.11	-1.82 ± 1.18	0.186
Executive functions	-4.66 ± 3.01	-2.35 ± 1.59	0.162
MMSE	17.29 ± 7.96	18.09 ± 5.61	0.625
CDR	1.09 ± 0.63	1.18 ± 0.65	0.670
ICV, mL	1762.83 ± 256.85	1583.37 ± 132.04	0.020
Mean WMH volume, mL (%)			
Frontal lobe	12.85 ± 29.15 (69.33%)	28.28 ± 15.0 (55.95%)	<0.001
Parietal lobe	4.45 ± 8.06 (24%)	16.97 ± 9.74 (33.57%)	<0.001
Temporal lobe	0.74 ± 1.78 (4%)	2.68 ± 1.92 (5.30%)	<0.001
Occipital lobe	0.49 ± 0.89 (2.67%)	2.62 ± 1.88 (5.18%)	<0.001

Abbreviations: SIVaD, subcortical ischemic vascular dementia; SD, standard deviation; MMSE, Mini-Mental State Examination; CDR, Clinical Dementia Rating; ICV, intracranial volume; WMH, white matter hyperintensity

*Fisher’s exact test

**Table 2:** Detailed clinical characteristics of included patients with *CSF1R*-related leukoencephalopathy.

Case no.	Sex	Age at onset	Age at MRI	Duration of disease at initial MRI (year)	Family history	Initial symptoms	Dementia	Psychiatric symptoms	Pyramidal signs	Parkinsonism	Seizure	CSF1R gene mutation
1	M	44	47	3	+	Bradykinesia	-	-	+	+	-	p.R782H
2	F	44	44	0	-	Optic ataxia	+	-	-	-	-	p.F849del
3	F	45	50	5	-	Bradykinesia, postural instability	+	+	-	+	-	p.P878S
4	M	47	49	2	+	Dysarthria, gait disturbance	-	-	-	+	-	p.A823V
5	F	51	52	1	-	Cognitive impairment	+	+	-	-	-	p.I794T
6	M	44	45	1	-	Cognitive impairment	+	+	-	+	-	p.G747* p.F971Serfs*7
7	M	51	55	4	-	Personality change	+	+	-	+	+	p.I794T
8	M	52	56	4	+	Personality change	+	+	-	+	+	p.C892_A894del
9	F	60	61	1	-	Cognitive impairment, gait disturbance	+	-	-	+	+	p.I794T
10	F	56	58	2	+	Bradykinesia	+	-	+	+	-	c.2442+5G>A
11	F	41	41	0	+	Gait disturbance, stuttering, hesitant speech	+	+	+	-	-	p.A781V
12	M	39	42	3	+	Tremor	+	+	-	+	-	pM766V
13	M	44	45	1	+	Cognitive impairment	+	+	+	-	-	p.A781V
14	F	39	40	1	+	Cognitive impairment	+	+	-	+	-	p.R782H

### Regional distributions of WMH in the *CSF1R*-related leukoencephalopathy and SIVaD groups

**Fig 1:**
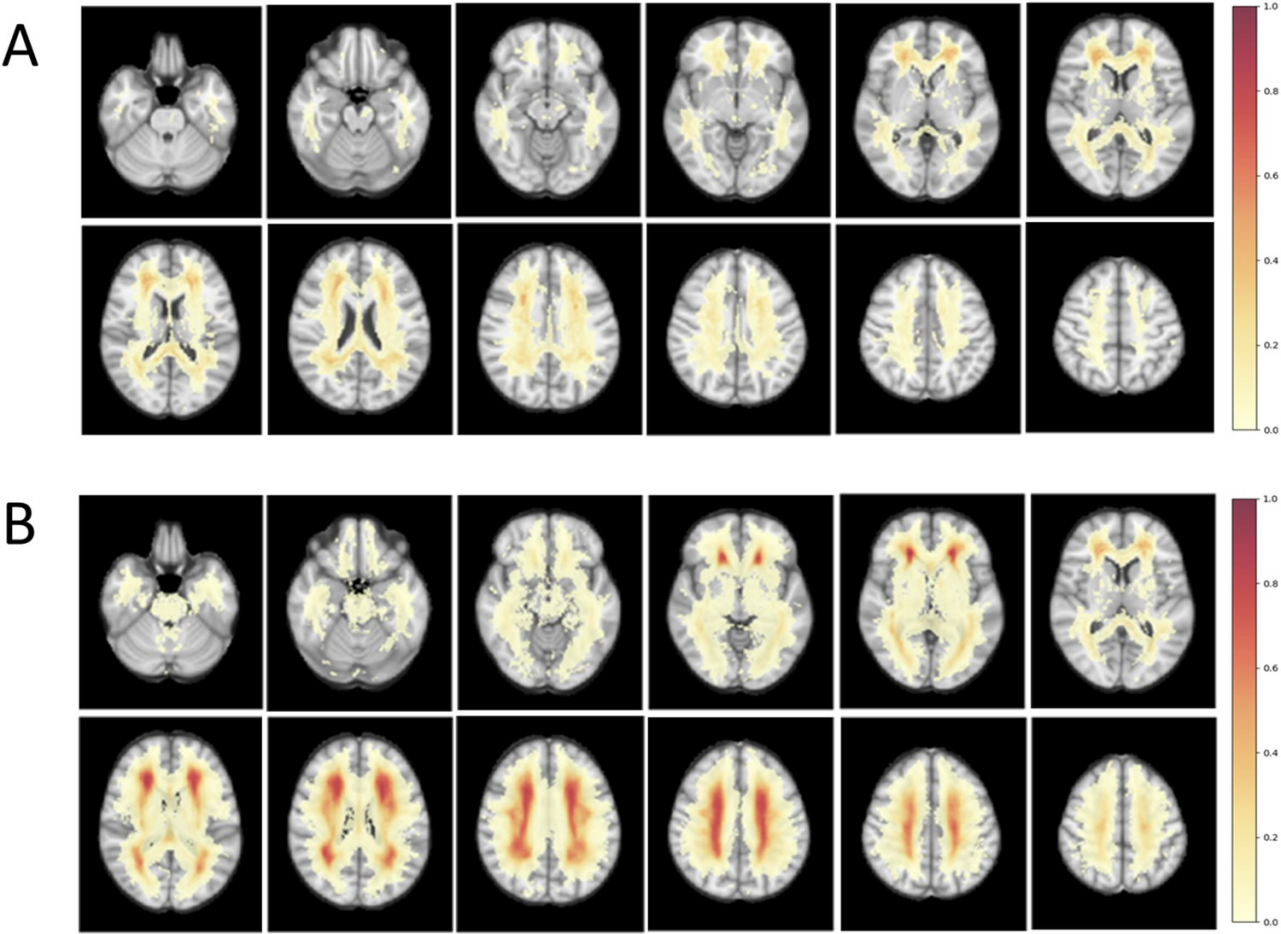
Comparison of the probability maps. It obtained after being overlaid WMH derived from FLAIR image of each patient with (A) *CSF1R*-related leukoencephalopathy and (B) SIVaD. The maps show WMH as color overlays (the color bar ranges from 0 to 1 reflecting the percentage of patients with WMH in each image voxel). Abbreviations: SIVaD, subcortical ischemic vascular dementia; WMH, white matter hyperintensity.

**Fig 2:**
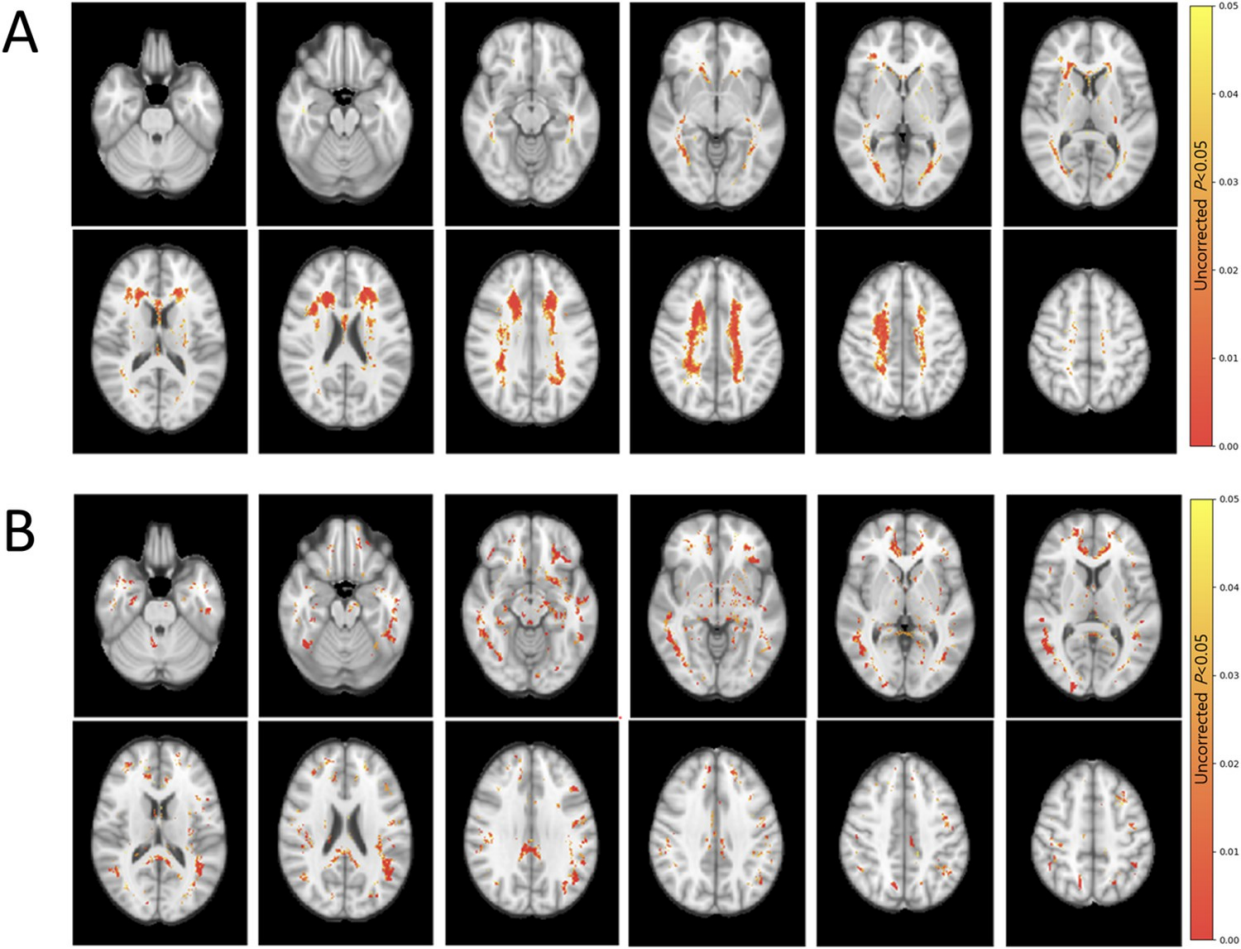
Topographical frequency maps of WMH in a single format. The color-overlaid areas represent more significant WMH in (A) the *CSF1R*-related leukoencephalopathy group than in the SIVaD group and (B) the SIVaD group than in the *CSF1R*-related leukoencephalopathy group. Abbreviations: WMH, white matter hyperintensity; SIVaD, subcortical ischemic vascular dementia.

Mean WMH volumes in the frontal, parietal, temporal, and occipital lobes were significantly higher in the SIVaD group than in the *CSF1R*-related leukoencephalopathy group (*p* < 0.001). Mean WMH volumes in the frontal and parietal lobes in both groups were higher than those in the temporal and occipital lobes (Table 1). In the probability map showing overlaid WMH from all patients in each group, the *CSF1R*-related leukoencephalopathy group tended to involve the bilateral frontal and parietal areas more than other areas, whereas the SIVaD group showed diffuse involvement in the bilateral frontal, parietal, and temporal areas (Fig 1). Spatial location and frequency maps based on group comparisons also demonstrated profound WMH in the bilateral frontal and parietal areas, with relatively spared temporal and occipital areas, in the *CSF1R*-related leukoencephalopathy group. In contrast, the SIVaD group showed more diffuse WMH involvement in the bilateral frontal, parietal, and temporal areas (Fig 2).

### Regional WMH and cognitive functions in the *CSF1R*-related leukoencephalopathy group

The WMH volume of any cerebral region in the *CSF1R*-related leukoencephalopathy group was not found to be associated with attention, language, visuospatial, memory, or executive function (Table 3).

**Table 3:** Effects of regional WMH on cognitive function in the *CSF1R*-related leukoencephalopathy group.

Regional WMH	Attention		Language and related function		Visuospatial function		Memory		Executive function	
	Average difference (SE)	*P*	Average difference (SE)	*P*	Average difference (SE)	*P*	Average difference (SE)	*P*	Average difference (SE)	*P*
Frontal lobe	0.09x10^-4^ (0.05x10^-3^)	0.848	0.03x10^-2^ (0.07x10^-2^)	0.674	-0.02x10^-2^ (0.08x10^-2^)	0.747	-0.01x10^-3^ (0.02x10^-2^)	0.963	0.03x10^-1^ (0.06x10^-1^)	0.636
Parietal lobe	0.02x10^-3^ (0.09x10^-3^)	0.815	0.04x10^-2^ (0.01x10^-1^)	0.766	-0.03x10^-2^ (0.02x10^-1^)	0.852	0.01x10^-2^ (0.04x10^-2^)	0.806	0.04x10^-1^ (0.04)	0.929
Temporal lobe	-0.05x10^-2^ (0.09x10^-2^)	0.588	0.04x10^-1^ (0.01)	0.769	-0.05x10^-1^ (0.02)	0.759	-0.02x10^-2^ (0.04x10^-1^)	0.973	0.18 (0.12)	0.240
Occipital lobe	0.04x10^-2^ (0.01x10^-1^)	0.727	0.04x10^-1^ (0.02)	0.821	-0.09x10^-1^ (0.02)	0.664	0.01x10^-1^ (0.05x10^-1^)	0.853	0.05 (0.14)	0.742

### Cortical thickness in the *CSF1R*-related leukoencephalopathy & SIVaD groups

**Fig 3:**
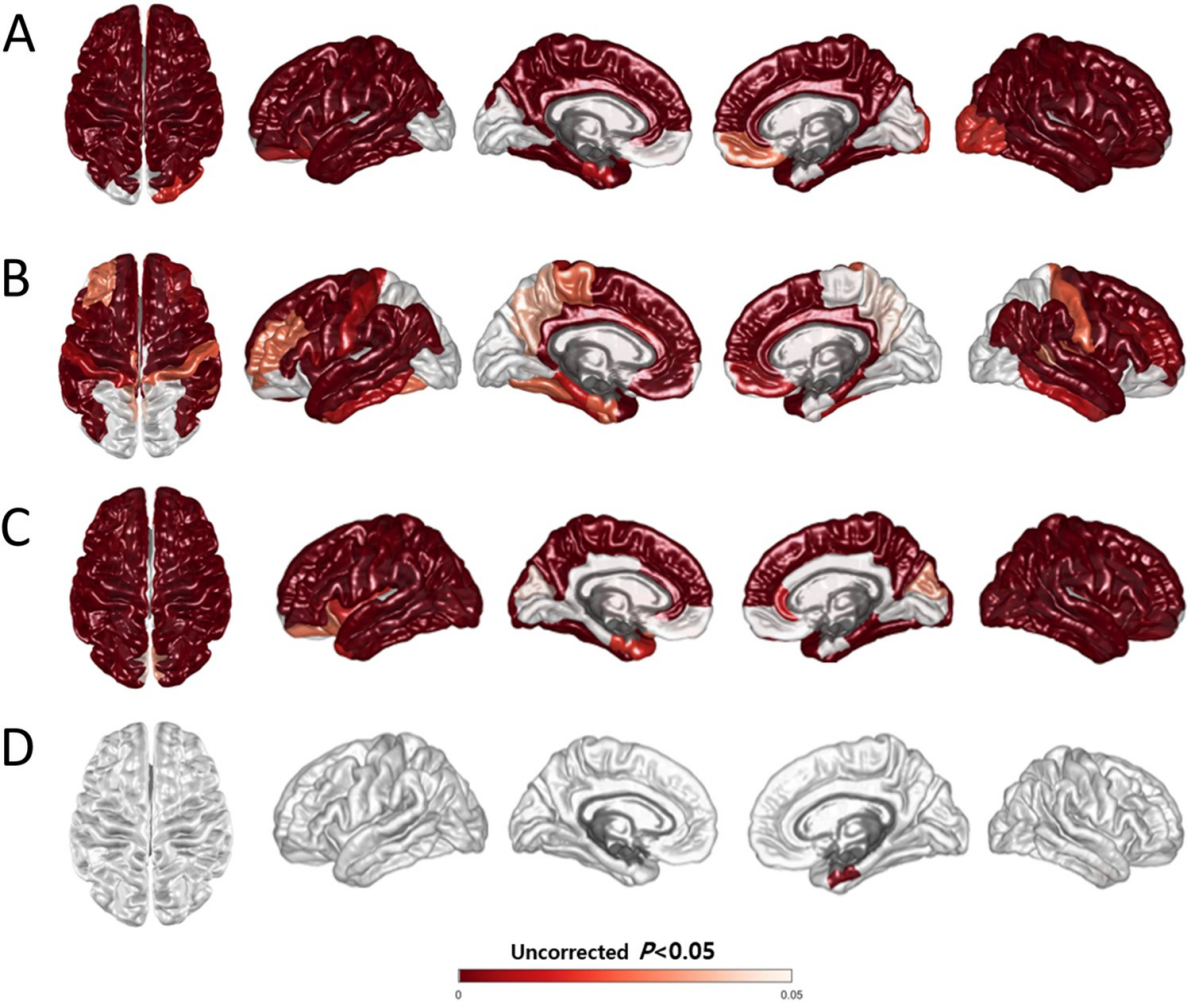
Three-dimensional statistical maps of cortical thickness. Colored areas represent regions with significant cortical atrophy in (A) the *CSF1R*-related leukoencephalopathy group compared with that in the CN group, (B) the SIVaD group compared with that in the CN group (C) the *CSF1R*-related leukoencephalopathy group compared with that in the SIVaD group, and (D) the SIVaD group compared with that in the *CSF1R*-related leukoencephalopathy group. The covariates were age, sex, education, and intracranial volume. Abbreviations: CN, cognitively normal; SIVaD, subcortical ischemic vascular dementia.

Compared with the CN group participants, patients in both groups showed widespread cortical thinning across the bilateral frontotemporoparietal areas, with relatively spared occipital regions (Fig 3A and 3B, Table 4), although the *CSF1R*-related leukoencephalopathy group showed more widespread cortical thinning than the SIVaD group (Fig 3C and 3D, Table 4).

**Table 4:** Comparison of cortical thickness between the *CSF1R*-related leukoencephalopathy and SIVaD groups.

	*CSF1R*-related leukoencephalopathy (*N* = 14)	SIVaD (*N* = 129)	CN (*N* = 121)	*p*-value	*CSF1R*-related leukoencephalopathy vs. SIVaD	*CSF1R*-related leukoencephalopathy vs. CN	SIVaD vs. CN
Frontal lobe, mm	2.19 ± 0.19	2.41 ± 0.14	2.47 ± 0.10	<0.001	<0.001	<0.001	<0.001
Parietal lobe, mm	2.00 ± 0.16	2.22 ± 0.13	2.25 ± 0.10	<0.001	<0.001	0.004	<0.001
Temporal lobe, mm	2.41 ± 0.26	2.57 ± 0.17	2.62 ± 0.12	<0.001	<0.001	<0.001	<0.001
Occipital lobe, mm	1.95 ± 0.15	1.96 ± 0.11	1.96 ± 0.11	0.052	0.012	0.104	0.519

Abbreviations: CC, corpus callosum; SIVaD, subcortical ischemic vascular dementia; CN, cognitively normal

Moreover, the *CSF1R*-related leukoencephalopathy group demonstrated greater cortical thinning across the entire cerebral cortex, except in the bilateral ventromedial prefrontal cortices, left parahippocampal gyrus, right entorhinal cortex, bilateral lingual gyri, and posterior cingulate cortex, compared with the SIVaD group (Fig 3C and Table 4). Cortical thickness in the right entorhinal cortex was significantly lesser in the SIVaD group than in the *CSF1R*-related leukoencephalopathy group (Fig 3D and Table 4).

### Corpus callosum in the *CSF1R*-related leukoencephalopathy and SIVaD groups

The mean volume of the CC in the *CSF1R*-related leukoencephalopathy group was significantly smaller than that in the SIVaD group (*p* = 0.007) and was the lowest in the genu. In contrast, the mean WMH volume in the CC in the *CSF1R*-related leukoencephalopathy group (2.86 ± 6.54 mL) tended to be larger than that in the SIVaD group (1.29 ± 0.61 mL), although the difference was not significant (*p* = 0.068). In the *CSF1R*-related leukoencephalopathy group, the mean WMH volume in the genu of the CC was the largest, followed by that in the body and splenium. In the SIVaD group, the mean WMH volume of body of the CC was the largest, followed by that of the genu and splenium. The regional proportion of WMH volume in the CC differed significantly between the *CSF1R*-related leukoencephalopathy and SIVaD groups (*p* < 0.001) (Table 5). The localized atrophy pattern of the CC on the *CSF1R*-related leukoencephalopathy compared with normal control differed from its regional WMH pattern; however, it did not differ between the *CSF1R*-related leukoencephalopathy and SIVaD groups.

**Table 5:** Comparison of corpus callosum volume with WMH volume.

	*CSF1R*-related leukoencephalopathy (*N* = 14)	SIVaD (*N* = 129)	CN (*N* = 121)	*p*-value^a^*
Mean CC volume, mL	0.38 ± 0.45	0.43 ± 0.03	0.46 ± 0.01	<0.001
Genu, mL	0.08 ± 0.01	0.90 ± 0.01	0.10 ± 0.00	<0.001
Body, mL	0.23 ± 0.03	0.25 ± 0.02	0.27 ± 0.01	<0.001
Splenium, mL	0.08 ± 0.01	0.09 ± 0.01	0.10 ± 0.00	<0.001
	*CSF1R*-related leukoencephalopathy (*N* = 14)	SIVaD (*N* = 129)	*p*-value^b^	
Mean WMH volume of CC, mL	2.86 ± 6.54	1.29 ± 0.61	0.068	
Genu (%)	67.78%	26.50%	<0.001	
Body (%)	16.42%	49.44%		
Splenium (%)	15.80%	24.06%		

Abbreviations: CC, corpus callosum; SIVaD, subcortical ischemic vascular dementia; CN, cognitively normal; WMH, white matter hyperintensity

a: Kruskal-Wallis test

b: Mann-Whitney U test

*Post hoc analyses showed significant differences between all three groups.

## Discussion

In this study, we characterized the differences in regional patterns of WMH and changes in cortical thickness between the *CSF1R*-related leukoencephalopathy and SIVaD groups using automated imaging techniques. The main findings of our study were as follows: (1) the *CSF1R*-related leukoencephalopathy group showed predominant subcortical white matter involvement of the bilateral frontoparietal areas, whereas the SIVaD group had more diffuse involvement of the bilateral frontoparietal and temporal regions; (2) thinning across almost the entire cortical region was greater in the *CSF1R*-related leukoencephalopathy group than in the SIVaD group; and (3) the CC was more atrophied and affected by WMH in the *CSF1R*-related leukoencephalopathy group than in the SIVaD group. Specifically, WMH in the *CSF1R*-related leukoencephalopathy group was most prominent in the genu of the CC. Overall, our findings provide new evidence regarding the different WMH distribution and cortical thinning patterns in *CSF1R*-related leukoencephalopathy compared with those in SIVaD.

Our first finding of predominant WMH in frontoparietal areas with a relatively spared temporal area in the *CSF1R*-related leukoencephalopathy group compared with that in the SIVaD group is in line with previous neuroimaging reports demonstrating prominent patchy or confluent and symmetric or asymmetric WMH in the frontal or frontoparietal areas in *CSF1R*-related leukoencephalopathy on visual inspection [6, 8, 23–25]. WMH on T2-weighted or FLAIR images in patients with *CSF1R*-related leukoencephalopathy has been pathologically confirmed as widespread white matter degeneration with loss of myelin and axons, abundant axonal spheroids, and pigmented macrophages [25–27]. According to the radiologic or pathologic staging of white matter lesions in *CSF1R*-related leukoencephalopathy, these lesions develop preferentially in the frontal or frontoparietal areas and extend to the occipitotemporal regions over time [28, 29]. As mean duration from onset to date of MRI of the patients in our study was 1.4 ± 1.7 years, which corresponds to early-stage disease considering the average disease duration of 6.8 ± 5.4 years [30], the finding of predominant WMH in the frontoparietal areas in our patients seems to be consistent with the temporal and spatial schemes of white matter lesions in *CSF1R*-related leukoencephalopathy. However, how and why *CSF1R* mutation triggers white matter degeneration in the frontal or frontoparietal areas remains unclear. Konno et al. recently suggested that *CSF1R*-related leukoencephalopathy is a primary microgliopathy, and that microglial dysfunction induced by *CSF1R* mutation is the primary cause of white matter degeneration [1]. Nevertheless, the pathophysiological links between aberrant microglia-mediated white matter degeneration and temporal/spatial neuropathological processes needs to be further elucidated. In comparison, the SIVaD group showed more widespread WMH without topographically specific patterns. WMH in SIVaD reflects partial demyelination, axonal loss, gliosis, and enlarged perivascular spaces, mainly induced by hypoxic stress or chronic ischemia caused by fibrohyalinotic or arteriolosclerotic vessel changes [31, 32]. A previous study using voxel-based approach showed the spatial distribution of WMH in elderly individuals depended on various vascular risk factors [33]. Therefore, WMH in our SIVaD group might be diffusely present in regions related to different pathogeneses or vulnerabilities based on patient-specific vascular risk factors [33–36]. Another possibility is that the SIVaD group was older and had a longer disease duration, which might have resulted in more diffuse WMH involvement [36, 37].

Second, although both groups showed significant cortical thinning throughout almost the entire cortex compared with that in CN controls, the thinning was more widespread in the *CSF1R*-related leukoencephalopathy group than in the SIVaD group. Cortical atrophy, one of the MRI characteristics of *CSF1R*-related leukoencephalopathy [29, 30], is usually associated with white matter lesions, but tends to appear as more generalized atrophy regardless of the localized pattern of WMH [38], which is in agreement with our results. Considering that neuropathological studies have shown that cortical neurons themselves are observed to be relatively spared, cortical atrophy or thinning in *CSF1R*-related leukoencephalopathy may be secondary to massive damage to both axons and myelin [38]. To date, no studies have reported cortical thinning patterns in *CSF1R*-related leukoencephalopathy. However, there has been one automated volumetric study comparing the brain volume fractions in *CSF1R*-related leukoencephalopathy with those in other diseases [39]. In that study, the authors revealed no significant differences of grey and white matter fractions between patients with *CSF1R*-related leukoencephalopathy and multiple sclerosis and speculated that their results could be attributable to a type II error, because the number of patients in each group was too small (n = 5). Another study exploring the imaging features of *CSF1R*-related leukoencephalopathy and cerebral autosomal dominant arteriopathy with subcortical infarcts and leukoencephalopathy (CADASIL) reported that predominant bifrontoparietal white matter lesions with cortical atrophy were more distinctive features of *CSF1R*-leukoencephalopathy than of CADASIL, whereas predominant temporal white matter lesions without cortical atrophy were more distinctive features of CADASIL than of *CSF1R*-leukoencephalopathy. However, the study also included only a small number of patients and used visual assessment of MRI scans [29]. In this regard, our study implemented more advanced methods and included relatively more patients, and thus may be more accurate and reliable than most previous studies that used visual ratings or scales and had smaller sample sizes.

Lastly, the *CSF1R*-related leukoencephalopathy group had a relatively smaller CC volume with more profound WMH, especially in the CC genu, than the SIVaD group. Although thinning of the CC with signal abnormalities is often observed in demyelinating diseases and other leukoencephalopathies [40–42], this is one of the most common MRI findings in *CSF1R*-related leukoencephalopathy [30] and has been included as one of the core features of the recently proposed diagnostic criteria for *CSF1R*-related leukoencephalopathy [43]. Similar to our study, Kinoshita et al. found that the CC index (CCI), determined based on the width and length of the CC, was significantly lower in *CSF1R*-related leukoencephalopathy than in VaD [44]. However, the CCI was manually calculated, and the WMH in the CC was not quantitatively measured; in addition, patients with VaD in that study were heterogeneous. Given that we applied automated quantification methods and our SIVaD group was rather homogeneous, our study and the study by Kinoshita et al. have limited comparability. Interestingly, we observed that the regional WMH pattern of the CC was not consistent with its localized CC atrophy pattern, but was consistent with the subcortical WMH pattern. This corresponds with the results of Sundal et al.’s study, which showed that WMH in the CC and CC atrophy (CCA) were not correlated with each other [38]. Regarding the underlying mechanism of CCA in SIVaD, the direct or indirect impact of vascular injury on subcortical white matter is believed to contribute to CCA, while “peculiar morphological alteration of microglia” has been suggested as the pathologic basis of CCA in *CSF1R*-related leukoencephalopathy [45].

The present study had a few limitations. First, the number of patients with *CSF1R*-related leukoencephalopathy was relatively small because of the rarity of this disease [8]. In addition, small number of patients made it difficult to identify the most discriminative MRI feature between the two groups. Second, most enrolled patients happened to have early-stage *CSF1R*-related leukoencephalopathy; it is possible that patients with a later stage of the disease would show more confluent WMH with severe cortical thinning on brain MRI, which may make it more challenging to distinguish *CSF1R*-related leukoencephalopathy from SIVaD. Finally, because genetic testing was not performed in patients with SIVaD, there might be a possibility that the patients with genetic leukoencephalopathy or genetic vascular leukoencephalopathy were included in the SIVaD group, even though they met the clinical diagnostic criteria for SIVaD. Despite these limitations, the strength of our study is that it is the first to use automated quantitative evaluations for WMH and cortical thinning in *CSF1R*-related leukoencephalopathy compared with those of SIVaD. Therefore, our findings can be expected to be more reliable than those based on the visual assessment methods used in most previous studies.

## Conclusion

In conclusion, we observed predominantly bilateral frontoparietal WMH patterns, profound cortical thinning, and CCA with WMH as unique MRI features of *CSF1R*-related leukoencephalopathy rather than SIVaD. To the best of our knowledge, this is the first study to use automated MR measurements to capture WMH, cortical thinning, and CCA with signal changes in *CSF1R*-related leukoencephalopathy. Our study highlights the capacity and utility of automated MRI technique for differentiating *CSF1R*-related leukoencephalopathy from other white matter diseases, even with a relatively small number of patients.

## Supporting information

S1 Table MRI sequence parameters for image acquisition and processing. (DOCX)
