# Supplementary material for: Distinct patterns of white matter hyperintensity and cortical thickness of CSF1R-related leukoencephalopathy compared with subcortical ischemic vascular dementia
Source: PLoS One. 2024 Oct 7;19(10):e0308989. doi: 10.1371/journal.pone.0308989 (PMC11458039; doi:10.1371/journal.pone.0308989)
Supplement: S1 Table — (DOCX) [file pone.0308989.s001.docx]

**Supplementary Table 1**

|  | Samsung Medical Center | | Pusan National University Hospital | | Pusan National University Yangsan Hospital | |
| --- | --- | --- | --- | --- | --- | --- |
| MRI scanner | Philips 3.0T Achieva, Netherlands | | Siemens 3.0T Skyra, Germany | | Siemens 3.0T Verio, Germany /  Magnetom 1.5T Avanto Germany | |
| Modality | T1 | FLAIR | T1 | FLAIR | T1 | FLAIR |
| Acquisition plane | Sagittal | Axial | Sagittal | Axial | Sagittal | Axial |
| Slice thickness, mm | 1.0 | 2.0 | 9.0 | 2.0 | 5.0 | 5.0 |
| Repetition time, ms | 9.9 | 11,000 | 2,000 | 10,130 | 2,000 / 498 | 9,000 / 9,000 |
| Echo time, ms | 4.6 | 125 | 2.29 | 81 | 9 / 10 | 94 / 81 |
| Flip angle, ˚ | 8 | 90 | 8 | 150 | 150 / 70 | 150 / 150 |
| Matrix size, pixels | 240 x 240 | 512 x 512 | 280 x 280 | 196 x 320 | 320 x 224 /  320 x 182 | 320 x 227 /  320 x 182 |

Abbreviation: FLAIR, fluid-attenuated inversion recovery
